# Prognostic value of pretreatment standardized uptake value of F-18-fluorodeoxyglucose PET in patients with gastric cancer: a meta-analysis

**DOI:** 10.1186/s12885-017-3271-z

**Published:** 2017-04-17

**Authors:** Zhonghua Wu, Junhua Zhao, Peng Gao, Yongxi Song, Jingxu Sun, Xiaowan Chen, Bin Ma, Zhenning Wang

**Affiliations:** Department of Surgical Oncology and General Surgery, The First Affiliated Hospital of China Medical University, 155 North Nanjing Street, Heping District, Shenyang, 110001 People’s Republic of China

**Keywords:** Gastric cancer, Positron emission tomography, Standardized uptake value, Prognosis, Meta-analysis

## Abstract

**Background:**

F-18- fluorodeoxyglucose Positron emission tomography (^18^FDG-PET) has been widely used in clinical practice. However, the prognostic value of the pretreatment standardized uptake value (SUV) for patients with gastric cancer remains controversial.

**Methods:**

Major databases were systematically searched. The quality of the included studies was assessed using the Newcastle–Ottawa scale; the PET protocols were also evaluated. The pooled hazard ratio (HR) for overall survival (OS) and recurrence-free survival (RFS) were used to estimate the effect size. Data from the included studies were analyzed using Review Manager Software version 5.2.

**Results:**

Eight studies with 1080 patients were included. The pooled HR for OS of six studies including 672 patients was 1.72 (95% CI [1.28–2.3], *p* = 0.0004, I^2^ = 0%), indicating that patients with high SUVs may have poor prognosis. The pooled HR for RFS was 1.70 (95% CI [1.20–2.39], *p* = 0.003, I^2^ = 0%). Subgroup analysis based on the cutoff values determining method indicated that the receiver operating characteristic (ROC) method could better define the cutoff value. Subgroup analysis based on the therapeutic strategies used subsequently indicated the significant prognostic value of SUV.

**Conclusion:**

In conclusion, our meta-analysis indicated that pretreatment SUV in primary lesions can be an important prognostic factor for overall survival and recurrence-free survival in patients with gastric cancer. High SUVs may indicate poor prognosis.

## Background

Gastric cancer is one of the most common types of cancer worldwide and is the second leading cause of cancer-related death, with approximately 700,000 deaths annually [1]. Although major improvements have been achieved in the early detection and screening of gastric cancer, many individuals are still diagnosed with advanced-stage gastric cancer every year, which underscores the poor prognosis of the disease [2]. Therefore, a practical method that can precisely predict the survival outcome of patients with gastric cancer is essential, because stratification of patients with potential survival outcomes could influence the treatment decision.

During the 1980s, positron emission tomography (PET) was incorporated into the clinical practice [3]. FDG-PET uses ^18^fluoro-deoxy-glucose (^18^F–FDG), a glucose analog, as tracer to evaluate the metabolic status of the morphological lesions. In order to quantify a lesion’s metabolic activity, standardized uptake value (SUV) is introduced to clinical practice. The SUV value provides a semi-quantitative analysis and description of the radioactivity in a lesion [4]. In practical work, a circular region of interest placed in the FDG-accumulating area was selected to obtain the SUV value. Because of the increased glycolytic activity of cancer cells, this imaging technique has been recently used for the detection of primary and metastatic lesions in the field of oncology, particularly in gastric cancer [5–9].

Furthermore, recent studies [10–13] have shown a significant relationship between prognosis and pretreatment PET imaging. This finding revealed that patients with a high standardized uptake value (SUV) had a worse prognosis than individuals with low SUV. This was confirmed in several types of cancer, including esophageal cancer and non-small cell lung cancer [10, 11]. However, some studies [4, 14–16] presented controversial conclusions for gastric cancer and a comprehensive analysis of the association between SUV and prognosis of gastric cancer have not yet been conducted. Therefore, this meta-analysis aimed to assess whether high SUV can be used as a prognosis predictor in patients with gastric cancer.

## Methods

### Literature search

We systematically searched the databases PubMed, EMBASE, the Cochrane library, and Web of Science for relevant articles from January 1975 to February 2016. We used the keywords “gastric cancer”,” stomach neoplasm”,” gastric carcinoma”,” stomach cancer”,” PET”,” positron emission tomography”,” 18F- FDG”,” 18-Fluoro-deoxy-glucose”, “F-18-fluorodeoxyglucose” and “2-Fluoro −2-deoxy-D-glucose” to summarize our search strategy. Moreover, we expanded our search by screening the references of relevant studies for additional studies that might be useful in our meta-analysis.

### Inclusion criteria and exclusion criteria

To keep our analysis accurate and reliable, we used the following inclusion criteria: (i) The studies reported at least one of the following outcome measures of interests: overall survival, recurrence-free survival and progression-free survival; (ii) a PET scan was performed prior to treatments, including chemotherapy, surgical therapy, and radiotherapy; (iii) studies only published in English with full-texts available were included. (iv) Studies contained a clear description of the PET protocol and reported the SUVmax or SUV mean of 18F–FDG. When several studies from the same authors or institutions were available, the meta-analysis included the most recent or highest-quality study.

Studies were excluded for the following reasons: (i) the prognostic information of patients was not reported in the studies; (ii) the hazard ratio (HR) could not be calculated considering the originally published data; (iii) the studies included patients diagnosed with gastro-esophageal junction carcinoma or gastrointestinal cancer; (iv) Studies were excluded if they only focused on the SUV of metastatic lymph nodes, surgical anastomoses or distant metastatic sites rather than primary tumor lesions.

### Data extraction and assessment of the study quality

Two investigators (Z.H. Wu and J.H. Zhao) independently reviewed the enrolled studies. Any discrepancies were presented to a third author and resolved through discussions among these investigators. The primary elements extracted consisted of the following: (1) the FDG avidity, which was defined as the focally increased 18F–FDG uptake exceeding the surrounding normal tissue [4]; (2) types of SUV, correction of SUVs, definition of threshold SUVs; (3) HR associated with the FDG uptake value for overall survival (OS), recurrence-free survival (RFS), progression-free survival (PFS), and their respective 95% confidence interval (CI). The main outcomes of our analysis were the pooled HRs for OS, RFS, and PFS.

We evaluated the quality of the enrolled studies according to the Newcastle–Ottawa scale (NOS) [17]. Studies that scored ≥7 of a maximum possible score of nine were regarded as high-quality trials whereas those scored ≥5 were recognized as moderate-quality trials. Furthermore, to systematically assess the methodological quality and ensure that the enrolled studies were accurate and reliable, we further evaluated them using a quality scale that was applied in a previous study [18]. This scale was composed of four categories: scientific design, generalizability, analysis of results and analysis of PET reports [18]. Each category contained several items, and each item was assigned values zero, one or two. And each category had a maximum score of 10 points.

### Statistical analysis

Review manager software version 5.2 (Cochrane Collaboration) was used to analyze the data collected from each study. To evaluate the prognostic effect representatively, we used the HR or estimated relative risk (RR) and their corresponding 95% CI as the effect variable. In cases in which we could not acquire the HR and its 95% CI explicitly, several relatively accurate methods reported by Tierney et al. [19] were used to calculate these values using data available in the literature.

The heterogeneity among the studies was calculated using the Cochrane Q-test and a value of I^2^ indicated the degree of heterogeneity [20]. In cases of lack of significant heterogeneity (I^2^ < 50%) among the studies, a fixed effect model was chosen for the meta-analysis [21]. Otherwise, a random effects model was used [21]. Publication bias was examined via the analysis of funnel plots [22]. In our meta-analysis, we calculated the pooled HR for OS, RFS, and PFS.

## Results

### Study selection and characteristics of the enrolled studies

**Fig. 1:**
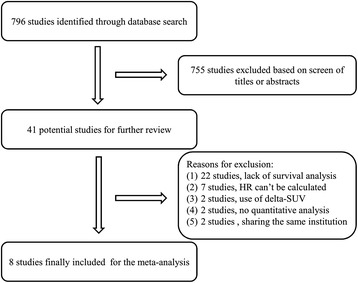
Flow diagram of study selection procedure

Using the aforementioned strategies, 796 relevant studies were identified. Among these, 755 studies were excluded after analysis of the titles or abstracts, mainly because they were reviews, case-control studies, cross-sectional studies, or not relevant to our analysis. After a careful analysis of the full texts of the remaining 41 articles, eight studies [4, 14–16, 23–26] were included in the meta-analysis. The detailed selection procedure is summarized in Fig. 1.

The eight studies evaluated involved 1040 patients. We excluded the studies in which the lesions (volume of interest) used to measure the SUV were defined in metastatic lymph nodes, surgical anastomoses, and distant metastatic sites. All eight studies presented the SUVs of primary lesions of gastric cancer and all reported pre-treatment values. Five studies [15, 16, 23, 24, 26] used maximum SUVs and two studies [4, 14] used SUVmean. Apart from these two measurements of SUV, one study [25] used the ratio between maximum and average SUV of normal livers. Among the eight included studies, six studies [4, 14, 16, 23, 25, 26] reported OS, two studies [25, 26] reported both OS and RFS, one study [16] reported PFS and OS, and two studies [15, 24] reported RFS and PFS respectively. For the determination of the cutoff values for high and low SUVs, five studies [15, 16, 24–26] used the receiver-operating characteristic (ROC), two studies [4, 14] used medians as the threshold values and one study [23] used averages as the cutoff values. The primary characteristics of the enrolled studies are presented in Table 1 and detailed information of the PET protocol is shown in Table 2.

**Table 1:** Primary characteristics of studies included in this meta-analysis

Study & author	Country	N Pts	FDG Avidity	Age	TNM Stage	Treatment	Endpoint	Type of SUV	SUV threshold definition	Threshold value	QS
Stahl 2003 [4]	Germany	40	60%	55 ± 10	T3-T4	Operation chemotherapy	OS	SUVmean	median value	4.6	57.90%
Mochiki 2004 [14]	Japan	85	75.20%	36–85	T1-T4 I–III	Operation	OS	SUVmean	median value	4	71.00%
Chung 2010 [23]	Korea	35	100%	57 ± 13	IV	Chemotherapy	OS	SUVmax	mean value	8	71.00%
Park 2012 [16]	Korea	82	100%	58.6 ± 12	I–IV	Inoperative palliative chemotherapy	OS and PFS	SUVmax	ROC	6	71.00%
Lee 2012 [15]	Korea	271	54.98%	60 ± 12	I–III	Operation	RFS	SUVmax	ROC	8.2	73.70%
Kim 2014 [24]	Korea	97	51.54%	59.8 ± 13.2	Ia-IV	Operation chemotherapy	PFS	SUVmax	ROC	5.74	71.00%
Lee 2015 [12]	Korea.	279	80%	20–93	pT2-pT4 pN0-pN4 IB–III	Operation	OS and RFS	TLR	ROC	2	65.80%
Song 2015 [26]	Korea	151	81%	58 ± 12.4	I–III	Operation	OS and RFS	SUVmax	ROC	4.5	71.00%

*Abbreviations*: *N pts.* number of patients, *OS* overall survival, *PFS* progression-free survival, *RFS* recurrence-free survival, *ROC* receiver-operating curve, *QS* quality scale, *TLR* the SUV of tumor/the SUV of normal liver tissue

**Table 2:** Characteristics of PET protocol

Study	NO. of patients	Patients with FDG-avid tumor	Correction of SUV	Blood glucose level(mg/dl)	Fasting time(h)	Uptake time(min)	Injected dose of FDG(MBq)	Type of the PET engine	Reconstruction method	Attenuation correction
Stahl 2003 [4]	40	24 (60%)	Body weight	unknown	6 h	40	300	Siemens	filter back-projection	ND
Mochiki 2004 [14]	85	64 (75.2%)	Body weight	unknown	4 h	40	275–370	SET 2400 W instrument	OS-EM	ND
Chung 2010 [23]	35	35 (100%)	Body weight	<120 NDM‚ <200 DM	6 h	Unknown	4.8/kg	GPS	LOR	CT
Park, 2012 [16]	82	82 (100%)	Body weight	≦130	4 h	60	unknown	GPS	OSEM and 3-D RAMLA	CT
Lee 2012 [15]	271	149 (55%)	Body mass	unknown	6 h	60	5.18 /kg	GPS	OS-EM	CT
Kim 2014 [24]	97	50 (51.5%)	Body weight	<130 NDM‚ <200 DM	6 h	50–60	3.7–5.5/kg	Biograph Truepoint 40	Unknown	CT
Lee 2015 [12]	279	223 (80%)	Body mass	unknown	4 h	60	370 for APS	Lee 2015 [12]	OS-EM	CT
Song2015 [26]	151	122 (81%)	Body weight	<150	6 h	60	5.5/kg	DPS	OS-EM	CT

*Abbreviations*: *NDM* no diabetes mellitus, *DM* diabetes mellitus, *OS-EM* ordered subset expectation maximization, *3-D RAMLA* row action maximum likelihood algorithm, *LOR* response algorithm, *ND* not determined, *APS* advanced pet scanner, *GPS* Gemini Philip scanner, *DSS* discovery ste scanner

### Quality assessment of the enrolled studies

The whole eight studies involving 1080 patients and the number of patients in each study ranges from 35 to 279. The quality assessment for the included studies using the NOS scale is displayed in Table 3. Among the eight studies, five studies had a score of six and three studies had a score of five and therefore were regarded as moderate-quality studies. The results of assessment of clinical and PET reports in each study are shown on Table 2. We applied the percentage of the full score to evaluate the quality of the studies. This percentage ranged between 57.9% and 73.7%, with a median of 71.0% (Table 1).

**Table 3:** The NOS quality of included studies

Study	Selection				Comparability		Outcome			Total	Quality
	REC	SNEC	AE	DO	SC	AF	AO	FU	AFU		
Stahl 2003 [4]	1	1	1	1	0	0	1	0	0	5	Moderate
Mochiki 2004 [14]	1	1	1	1	0	0	0	0	1	5	Moderate
Chung 2010 [23]	1	1	1	1	0	0	0	1	1	6	Moderate
Park 2012 [16]	1	1	1	1	0	0	1	0	0	5	Moderate
Lee 2012 [15]	1	1	1	1	0	0	0	1	1	6	Moderate
Kim 2014 [24]	1	1	1	1	0	0	0	1	1	6	Moderate
Lee 2015 [12]	1	1	1	1	0	0	1	1	0	6	Moderate
Song 2015 [26]	1	1	1	1	0	0	0	1	1	6	Moderate

*REC* representativeness of the exposed cohort, *SNEC* selection of the non-exposed cohort, *AE* ascertainment of exposure, *DO* demonstration that outcome of interest was not present at start of study, *SC* study controls for age, sex, *AF* study controls for any additional factors (chemoradiotherapy, curative resection), *AO* assessment of outcome, *FU* follow-up long enough (36 M) for outcomes to occur, *AFU* adequacy of follow-up of cohorts. “1” means that the study is satisfied the item and “0” means the opposite situation

### Prognostic value of SUV for overall survival

**Fig. 2:**
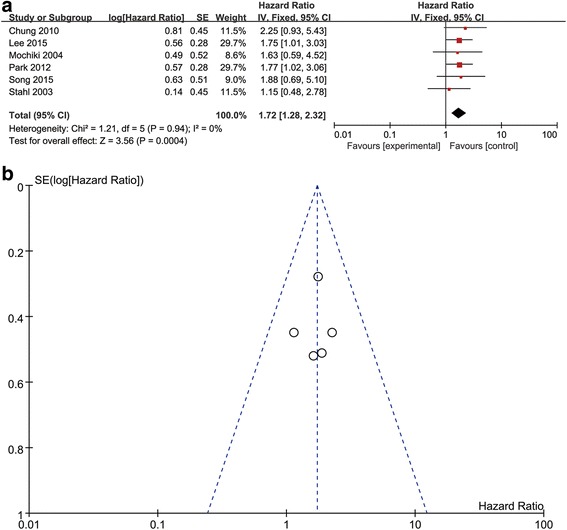
**a** Forest plot of HR for overall survival. **b** Test result for publication bias

Six of the eight studies were selected to acquire the pooled HR for OS. Among the six studies for OS, 672 patients were included. Within the 672 included patients, 550 patients were diagnosed with FDG-avid gastric tumor. To assess the prognostic value of SUV, a meta-analysis was performed on the six studies that reported the OS. The analysis of these studies using the fixed-effect model indicated that the pooled HR for OS was 1.72 (95% CI [1.28–2.32], *P* = 0.0004, I^2^ = 0%) (Fig. 2a), revealing that high SUVs were significantly associated with poorer prognosis. Meanwhile, there was no evidence of publication bias according to the funnel plot (Fig. 2b).

**Fig. 3:**
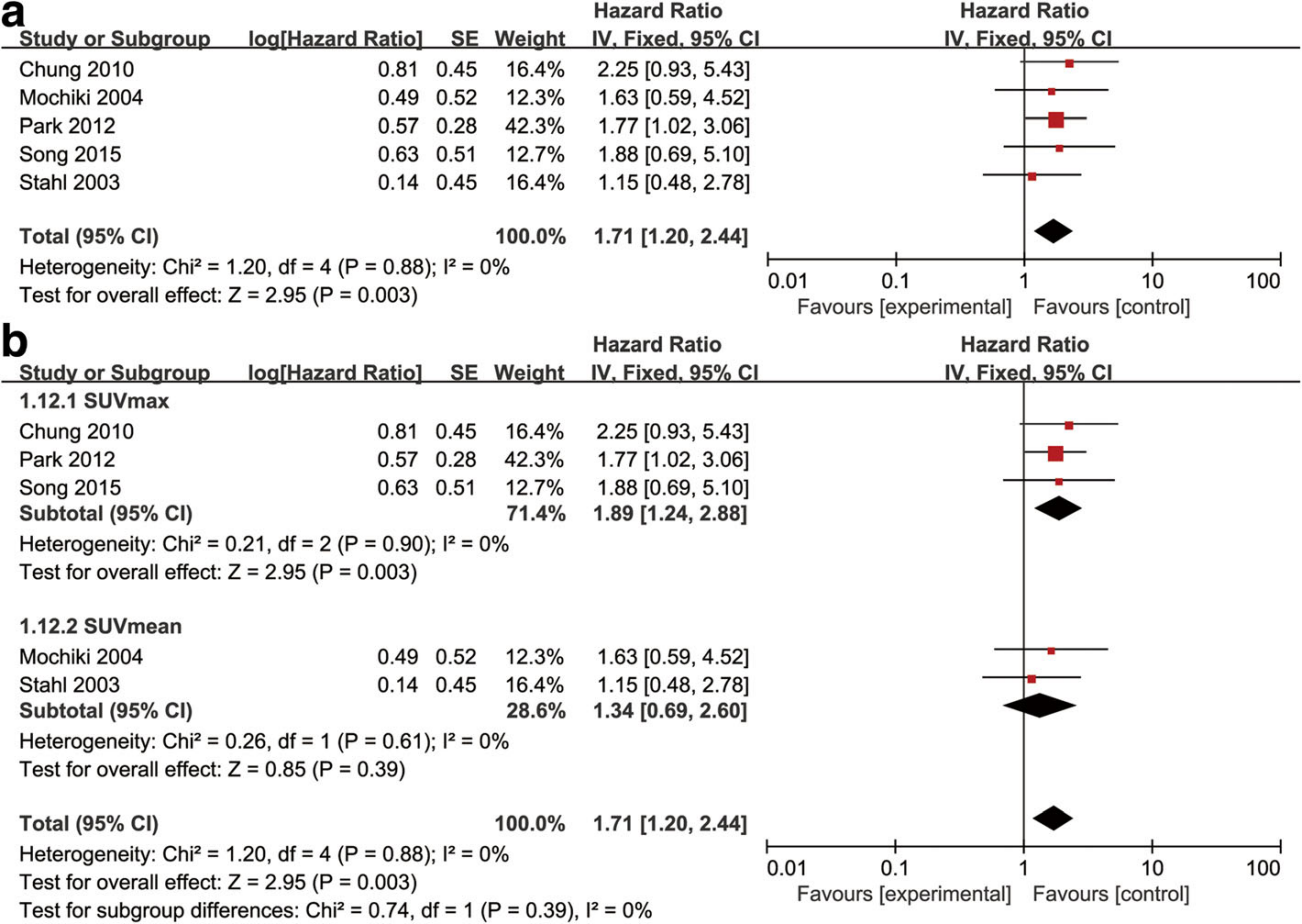
**a** The results of sensitivity analysis after excluding the study using SUV ratio. **b** The results of subgroup analysis based on SUVmax and SUVmean

As there is one study using the SUV ratio between lesion and normal liver parenchyma, we performed sensitivity analysis removing this study to investigate the effect of SUV values on prognosis predicting. Results of sensitivity analysis was in accordance with the result of meta-analysis included the SUV ratio and showed that SUV values can be a prognostic factor for prognosis (HR = 1.71, 95% CI [1.20–2.44], *P* = 0.003, I2 = 0%) (Fig. 3a). In additon, we performed a subgroup analysis based on SUV types, the result of subgroup analysis indicated that high SUV values held a significant prognostic effect in SUVmax subgroup (HR = 1.89, 95% CI [1.24–2.88], *P* = 0.003, I2 = 0%) but not in the SUVmean subgroup (HR = 1.34, 95% CI [0.69–2.60], *P* = 0.39, I2 = 0%) (Fig. 3b).

**Fig. 4:**
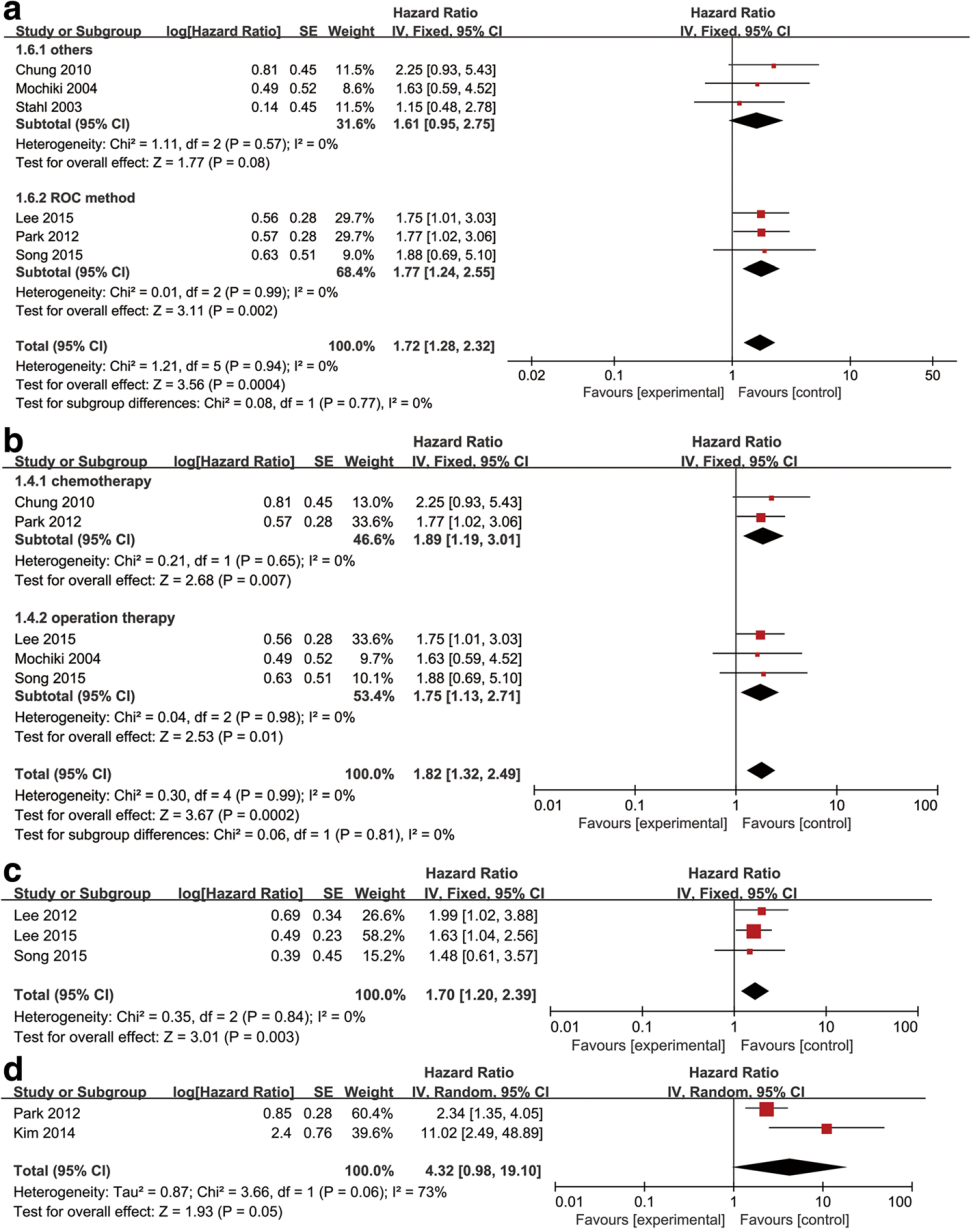
**a** Subgroup analysis based on cutoff value determining methods. **b** Subgroup analysis based on subsequent therapy strategies. **c** Forest plot of HR for reference-free survival. **d** Forest plot of HR for progression-free survival

Subsequently, we performed a subgroup analysis using the methods that provided the cutoff values. As shown in Fig. 4a, a significant prognostic value for high SUV was found in the subgroup for which the cutoff value was determined using ROC curves (HR = 1.77, 95% CI [1.24–2.55], *P* = 0.0002, I^2^ = 0%) but not in the subgroup for which the cutoff value was determined using other methods (HR = 1.61, 95% CI [0.95–2.75], *P* = 0.08, I^2^ = 0%).

Furthermore, a subgroup analysis was performed on the basis of the categories of therapies used subsequently. The results (Fig. 4b) indicated that high SUVs reflected poor prognosis in both the subgroups (overall: HR = 1.82, 95% CI [1.32–2.49], *P* = 0.0002, I^2^ = 0%; chemotherapy subgroup: HR = 1.89, 95% CI [1.19–3.01], *P* = 0.007, I^2^ = 0%; surgery subgroup: HR = 1.75, 95% CI [1.13–2.71], *P* = 0.01, I^2^ = 0%).

### Prognostic value of SUV for progression-free survival and recurrence-free survival

A meta-analysis was also performed on the three studies that reported the RFS. The pooled HR was 1.70 (95% CI [1.20–2.39], *p* = 0.003, I^2^ = 0%) indicating that higher SUVs were correlated with the poor prognostic effect of RFS (Fig. 4c). PFS was determined considering data from two studies. The results obtained from the random effect model indicated that high SUV had no significant predictive value on PFS (HR = 4.32, 95% CI [0.98–19.10], *p* = 0.05, I2 = 73%) (Fig. 4d).

## Discussion

In recent decades, FDG-PET has been widely used in clinical practice for staging patients with cancer and for detecting local and distant metastasis [27–30]. In recent years, several studies [12, 26] in the field of gastric cancer have focused on the prognostic value of metabolic activity detected by pretreatment FDG-PET. However, whether the SUV of primary tumors is a prognostic factor in patients with gastric cancer remains unclear. Some studies [16, 25] that investigated the prognostic value of SUV in gastric cancer found a significant prognostic value of high SUV whereas other studies [4, 14] did not find any evident relationship between SUV and prognosis.

A meta-analysis was the statistical pooling of the outcomes identified in individual studies. Therefore, it can increase the precision of the estimated effect of the individual studies and consequently elucidate the relationship between the observed variables and the outcomes and can eventually be applied in clinical practice [31]. In the present meta-analysis, the analysis of the pooled HR for OS indicated that patients with a high SUV had higher risk of death than those with low SUV on the basis of the threshold values. Moreover, we found that high SUV was an important factor for predicting RFS. These findings are important because this is the first meta-analysis of studies with controversial opinions on the prognostic value of SUV in primary lesions of gastric cancer. To date, patient characteristics like tumor size, cancer staging, and the status of local or distant metastasis have been widely acknowledged as significant prognostic factors for gastric cancer [31–34]. As SUV values held an advantage that they can reflect the metabolic status of lesion compared with other diagnostic methods, SUV and patient characteristics can be synergistically used to predict prognosis. Meaningfully, a previous study [26] showed combining SUV value and pT stage could increase the value of SUV for predicting prognosis. Therefore, our study provided a direction towards studying on the prognostic role of combining SUV and patients characteristics like pT stage or others.

The standardized uptake value (SUV) was introduced for quantitative analysis. For calculating the SUV value, regions of interest were selected from primary tumor lesion in the trans-axial PET image where the lesion seemed to have the most intense FDG uptake. In that way, SUVmean represents the mean value of the SUVs within the selected regions of interest and SUVmax is the largest value among the SUVs of the selected regions of interest. And both SUVmean and SUVmax can reflect the SUV values of the tumor lesions. Our subgroup analysis based on SUV types indicated that there is a significant relationship between high SUV and poor prognosis in the subgroup applying the SUVmax as the SUV value of tumor lesion. However, in the subgroup using the SUVmean, the relationship is not significant. This can be explained by that when using the SUVmean, it is more likely for us to neglect the larger SUVs among the regions of interest. This outcome also reminded us that we should give priority to SUVmax when designing a study focusing on the SUV value and prognosis.

The enrolled studies used several methods to determine the threshold SUV. Some studies [4, 14, 23] used the median or mean value (other methods) as the cutoff value because they argued that the ROC method tended to generate many false-positive results. Other studies [15, 16, 24–26] used ROC curves. In addition, the results of the subgroup analysis indicated that, in the studies that applied the ROC method, the patients with high SUV had a pooled 1.77-fold higher risk of death (Fig. 2b) whereas the studies that used other methods found no significant relationship between SUV and prognosis. Moreover, a study suggested that the ROC method could help identify the most appropriate threshold value [35].

In the current stage, because of inconsistent PET techniques, use of different PET protocols, and differences in patient characteristics depending on the geographical region evaluated, it was extremely difficult for different medical centers to find a consistent threshold value to distinguish patients with high or low SUV. Previous studies pointed out that a value of 2.5 could be used as the cutoff value for tumor delineation [36–39]. The threshold values in the studies evaluated herein varied between 4.6 and 8.2, indicating that the ROC method was the ideal method to determine the cutoff value; therefore, these studies provided a strategy that allowed the ROC method to be consistently used to calculate the cutoff value. Our study provides a direction and evidence that SUV value is a potential parameter for prognosis predicting. In this respect, the obtaining of a consistent threshold value from all the available studies will better serve the prediction of the prognosis in the future. Therefore, to obtain an accurate standard threshold values in clinical practice, further studies are needed to formulate a systemic PET protocol assessing standard to get a consistent cutoff value and eventually promote the utilization of SUV in predicting prognosis.

During the selection of eligible studies, we chose the studies that investigated disease prognosis and determined the pretreatment SUV. In this manner, the treatment strategies used subsequently in the patients included in these studies were noteworthy because these therapies played a key role in determining prognosis. Our subgroup analysis based on these treatment strategies suggested that high SUV was associated with poor prognosis in both the subgroups and indicated that the prognosis-predicting value of pretreatment SUV was not affected by the subsequent therapies used in patients with gastric cancer.

The analysis of the full texts of the included studies indicated that, in some studies, the authors excluded the patients with a history of diabetes mellitus [4, 14]. Previous studies on bronchial and cervical cancer pointed out that diabetes had no significant influence on the uptake of FDG [40, 41]. However, in other studies [42, 43], a long-term high level of blood glucose had an impact on the uptake of FDG, thereby affecting its detection via PET. Furthermore, some studies [44–46] suggested that pre-existing diabetes mellitus increased the risk of gastric cancer, which indicates that many patients with gastric cancer may also present with diabetes mellitus. Unfortunately, to date, no published studies have focused on the influence of diabetes on PET performance in patients with gastric cancer. Therefore, further studies are needed to elucidate this influence.

This is the first meta-analysis that evaluated the prognostic value of SUV in patients with gastric cancer. We acknowledge some limitations in our study. First, the number of studies included in our meta-analysis was relatively small and eight papers included this meta-analysis were regarded as moderate quality according to NOS. We expect that more high-quality studies on this issue will be published in the future. Second, because of the low morbidity observed in Western countries, most studies were confined to eastern Asia, which could decrease the representativeness of our results for gastric cancer on a global scale. In addition, we hope that more studies from different centers and different geographical regions will be carried out in the future. Third, as mentioned above, we were unable to find a fixed cutoff value to distinguish the patients with high or low SUV. Therefore, more studies with data from individual patients are needed to obtain standard threshold values for predicting the prognosis of gastric cancer. However, despite these limitations, this is the first meta-analysis that evaluated the prognostic value of SUV in gastric cancer. We found that patients with high pretreatment SUV tended to have poor prognosis. Furthermore, our results suggested that the ROC method could better define a threshold value.

## Conclusion

In conclusion, our meta-analysis indicated that pretreatment SUV in primary lesions can be an important prognostic factor for overall survival and recurrence-free survival in patients with gastric cancer. High SUVs may indicate poor prognosis.

## Abbreviations

^18^FDG-PET: F-18-fluorodeoxyglucose Positron emission tomography
SUV: standardized uptake value
NOS: Newcastle–Ottawa scale
ROC: receiver operating characteristic
GC: gastric cancer
HR: hazard ratio
OS: overall survival
RFS: recurrence-free survival
PFS: progression-free survival
